# Real-Time Colorimetric Imaging System for Automated Quality Classification of Natural Rubber Using Yellowness Index Analysis

**DOI:** 10.3390/jimaging11110397

**Published:** 2025-11-07

**Authors:** Suphatchakorn Limhengha, Supattarachai Sudsawat

**Affiliations:** 1 Faculty of Sciences and Industrial Technology, Prince of Songkla University, Surat Thani Campus, Surat Thani 84000, Thailand; suphatchakorn.l@psu.ac.th; 2 Department of Materials Handling and Logistics Engineering, Faculty of Engineering, King Mongkut’s University of Technology North Bangkok, Bangkok 10800, Thailand

**Keywords:** colorimetric imaging, image processing, color classification, quality control, natural rubber, CIELAB color space, yellowness index, computer vision

## Abstract

Natural rubber quality assessment traditionally relies on subjective visual inspection, leading to inconsistent grading and processing inefficiencies. This study presents a colorimetric imaging system integrating 48-megapixel image acquisition with automated colorimetric analysis for objective rubber classification. Five rubber grades—white crepe, STR5, STR5L, RSS3, and RSS5—were analyzed using standardized 25 × 25 mm^2^ specimens under controlled environmental conditions (25 ± 2 °C, 50 ± 5% relative humidity, 3200 K illumination). The image processing pipeline employed color space transformations from RGB through CIE1931 XYZ to CIELAB coordinates, with yellowness index calculation following ASTM E313-20 standards. The classification algorithm achieved 100% accuracy across 100 validation specimens under controlled laboratory conditions, with a processing time of 1.01 ± 0.09 s per specimen. Statistical validation via one-way ANOVA confirmed measurement reliability (*p* > 0.05) with yellowness index values ranging from 8.52 ± 0.52 for white crepe to 72.15 ± 7.47 for RSS3. Image quality metrics demonstrated a signal-to-noise ratio exceeding 35 dB and a spatial uniformity coefficient of variation below 5%. The system provides 12-fold throughput improvement over manual inspection, offering objective quality assessment suitable for industrial implementation, though field validation under diverse conditions remains necessary.

## 1. Introduction

Natural rubber represents a critical agricultural commodity with global annual production exceeding 14 million tons, serving industries from automotive manufacturing to medical devices. Quality assessment through color evaluation directly influences market value, with premium light-colored grades commanding prices 30–50% higher than standard grades in international markets [1,2]. Traditional quality control, however, relies predominantly on subjective visual inspection under variable lighting conditions, resulting in classification inconsistencies between operators and facilities [3,4]. This inherent variability creates significant challenges for international trade, where objective quality documentation is essential for price negotiation and dispute resolution.

Color development in natural rubber reflects biochemical processes, including protein oxidation and impurity incorporation during processing, making quantitative color assessment a reliable quality indicator [5,6]. The darkening process, influenced by temperature, humidity, and exposure time, directly impacts end-product quality [7]. The yellowness index, standardized by ASTM E313-20, provides a quantitative metric for chromatic deviation from ideal whiteness with established correlations to rubber quality grades [8]. Computer vision and colorimetric imaging have revolutionized agricultural quality control by enabling rapid, non-destructive assessment [9,10]. Previous rubber classification studies achieved 85–97.5% accuracy using color indices [11], neural networks [12,13], and hyperspectral imaging [14], but limitations in environmental control, processing speed, and standardization have prevented widespread adoption. Our previous work demonstrated the feasibility of automated color analysis but identified needs for real-time processing and comprehensive validation [15].

This study addresses these limitations by developing an integrated colorimetric imaging system combining high-resolution acquisition under controlled conditions with efficient image processing algorithms. Primary objectives include establishing reproducible imaging protocols, validating the yellowness index as a reliable metric, demonstrating industrial-scale classification accuracy, and characterizing system performance through quantitative metrics.

## 2. Materials and Methods

### 2.1. Sample Preparation and Specimen Characteristics

Five distinct natural rubber types representing diverse quality grades were selected for comprehensive analysis, encompassing the spectrum of commercially significant rubber grades used in international trade. White crepe represents a premium grade characterized by minimal color development and rapid processing from fresh latex. Standard Thai Rubber grades 5 and 5L (STR5, STR5L) represent mid-grade rubbers processed from field latex, with STR5L exhibiting reduced yellowing due to optimized processing conditions. Ribbed Smoked Sheet grades 3 and 5 (RSS3, RSS5) represent progressively darker grades subjected to controlled smoking treatments. Raw rubber materials obtained from certified producers following Standard Thai Rubber specifications underwent standardized processing to ensure material uniformity and optical consistency [16]. Coagulation control employed formic acid (0.5% *v*/*v*) at ambient temperature for 12–16 h, selected over sulfuric acid to minimize color alteration [17]. Coagulated rubber was processed using a laboratory two-roll mill applying a consistent pressure of 5.0 MPa with a mill gap set at 1.70 mm to achieve the target thickness, with each specimen undergoing three compression passes to ensure uniform density [18]. Compressed sheets were folded into two layers (final thickness 3.40 mm) and cut into 25 × 25 mm^2^ squares, providing sufficient area for spatial analysis while fitting within the microscope field of view. Twenty specimens per rubber type (n = 100 total) were prepared according to ISO 2393:2017 standards [16], processed within 24 h of coagulation, and analyzed within 48 h to prevent oxidation-induced color alterations [19]. Specimens were stored in sealed containers at 25 °C and 50% relative humidity between processing and imaging. The sample size of n = 20 per rubber type (100 total) was determined through statistical power analysis targeting the detection of 5-unit yellowness index differences with power = 0.90 and *α* = 0.05. This meets ISO 5725-2:2019 requirements [20] (minimum n = 15/condition) for precision studies. Specimens were freshly processed under standardized protocols to validate measurement accuracy without confounding factors from degradation or contamination. Industrial validation will require evaluation of degraded specimens (aged 1 week to 6 months), contaminated samples (dust, moisture, residual chemicals), and edge-quality materials representing production variability.

**Figure 1:**
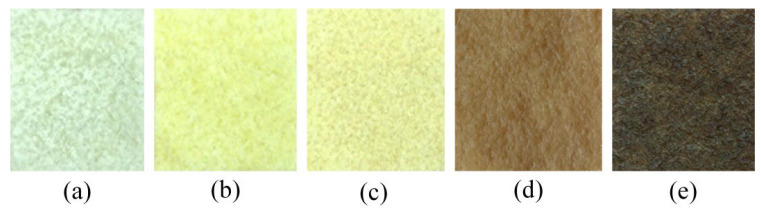
Natural rubber specimens prepared for colorimetric imaging analysis: (**a**) white crepe rubber exhibiting the highest lightness ($L^{*}$ = 51.48, YI = 8.52); (**b**) Standard Thai Rubber grade 5 (STR5) with intermediate yellowness ($L^{*}$ = 46.29, YI = 62.84); (**c**) STR5L showing slight greenish undertone ($L^{*}$ = 46.91, $a^{*}\;$= −0.88, YI = 64.31); (**d**) Ribbed Smoked Sheet grade 3 (RSS3) with highest yellowness index ($L^{*}$ = 26.51, YI = 72.15); (**e**) RSS5 with darkest appearance ($L^{*}$ = 8.86, YI = 28.79). All specimens were 25 × 25 mm^2^ with a thickness of 1.70 ± 0.1 mm, processed using standardized compression to ensure uniform optical properties.

Figure 1 illustrates the five prepared rubber types showing distinct color gradations corresponding to quality grades, ranging from white crepe exhibiting the highest lightness through progressive darkening to RSS5 with the lowest lightness value. The visible color progression reflects the biochemical and processing differences that characterize each grade, validating the need for objective quantitative assessment methods.

### 2.2. Automated Imaging System Design and Calibration

The automated imaging system was designed to replace conventional light box inspection with a fully integrated, computer-controlled platform, eliminating operator-dependent variability. The system comprises five functional modules working in concert to provide consistent, reproducible colorimetric measurements. The computational processing unit utilizes an Intel Core i7-11700 processor with 16 GB RAM and dedicated SSD storage, with GPU acceleration employed for color space transformations and matrix operations, reducing processing time by approximately 40% compared to CPU-only computation. The high-resolution imaging module employs a digital microscope with a CMOS sensor (8000 × 6000 pixels, 14-bit color depth per channel), providing 48 megapixel resolution with variable magnification up to 180×, operating at a working magnification of 20× to provide 0.5 mm spatial resolution across the specimen field. The controlled illumination array incorporates custom-designed LED arrays with six high-CRI white LEDs (CRI > 90) positioned in a circular arrangement at 45° incident angles, minimizing specular reflection while ensuring uniform illumination across the specimen surface. Luminous flux is adjustable from 180 to 720 lux at the specimen plane, with working conditions set at 1500 lux for optimal sensor utilization without saturation. The environmental control chamber maintains enclosed imaging conditions (500 × 500 × 400 mm^3^) with active temperature control at 25 ± 2 °C using thermoelectric cooling elements and humidity regulation at 50 ± 5% relative humidity via desiccant cartridges and a humidification system, with HEPA filtration preventing particulate contamination of optical surfaces. The automated specimen handling system employs a motorized X-Y positioning stage with 10 μm repeatability and vacuum hold-down, preventing specimen movement during acquisition.

**Figure 2:**
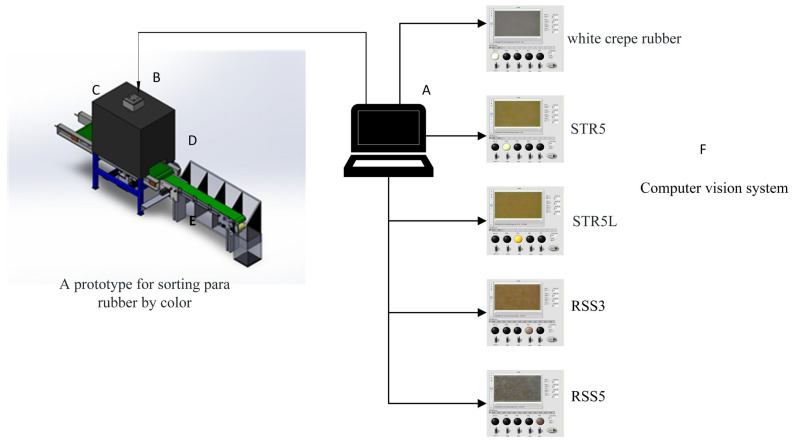
Automated color classification system for Para rubber quality assessment: (A) computational processing unit (Intel Core i7-11700, 16 GB RAM) for real-time image analysis; (B) high-resolution digital microscope (48 MP, 180× magnification) with achromatic optics; (C) adjustable LED illumination arrays (3200 K, CRI > 90) positioned at 45° for uniform specimen lighting; (D) environmental control chamber maintaining 25 ± 2 °C and 50 ± 5% humidity with HEPA filtration; (E) automated specimen positioning platform with precision alignment; (F) real-time classification interface displaying colorimetric analysis results. The modular design enables component replacement and system calibration without extended downtime.

Figure 2 presents the complete integrated system architecture, demonstrating the modular design that facilitates maintenance and calibration procedures. LED spectral distribution was characterized using a calibrated spectroradiometer (USB4000, Ocean Optics Inc., Dunedin, FL, USA; 0.5 nm resolution) across 350–800 nm, revealing a close match to CIE standard illuminant D65 with correlation coefficient R^2^ = 0.9987, mean absolute spectral difference of 0.34%, and maximum deviation of 0.6% at 460 nm characteristic of white LED phosphor conversion. Complete spectral power distribution data comparing measured LED emission spectrum against D65 standard across 21 wavelength points from 380 to 780 nm is presented in Figure S1 (Supplementary Materials), demonstrating excellent agreement throughout the visible and near-infrared spectrum with all deviations remaining below ±0.7%. The correlated color temperature of 6489 K differs from D65 by only 15 K, confirming spectral equivalence for colorimetric applications. Color rendering index calculated following CIE 13.3 methodology yielded a CRI of 92.5, exceeding the threshold recommended for critical color evaluation applications. Illumination uniformity was quantified by imaging white reference tiles and calculating the spatial coefficient of variation, yielding a CV of 3.2% across a 30 × 30 mm^2^ central region, meeting stringent requirements for colorimetric imaging. System calibration procedures included geometric calibration using precision grid targets to correct barrel distortion of 0.28% at image edges, radiometric calibration employing certified reflectance standards (Spectralon SRT-99-100, 99% reflectance) for white balance and flat-field correction, and colorimetric calibration validated using X-Rite ColorChecker Classic (X-Rite Inc., Grand Rapids, MI, USA). Measured CIELAB coordinates compared against manufacturer-certified values yielded a mean color difference ∆Eab of 1.18 ± 0.31 units across all 24 patches, with maximum deviation of 1.52 units for the red patch (Patch 15). Complete calibration of the validation data, including reference versus measured L, $a^{*}$, and $b^{*}$ values, scatter plots showing correlation ($R^{2}$ = 0.9998 for ${\;L}^{*}$ values), chromatic coordinate comparison in CIELAB space, and bar chart of ∆Eab color differences for all 24 patches is presented in Figure S2 (Supplementary Materials), confirming all measurements fell within acceptable tolerances (∆Eab < 2.0) for quality control applications with 100% of patches meeting acceptability criteria.

### 2.3. Image Acquisition Protocol and Processing Pipeline

Specimens were placed in the imaging chamber for 5 min of thermal equilibration to chamber conditions before image acquisition. Automated positioning stage centered specimens within the imaging field using fiducial markers detected via corner detection algorithms. LED arrays were powered for 15 min of warm-up before initiating measurements, based on temporal stability characterization showing minimal flux variation after this period. Three sequential images were acquired per specimen at 1 s intervals with exposure times automatically adjusted to utilize 60–80% of sensor dynamic range, preventing saturation while maximizing signal-to-noise ratio. For the five rubber types, optimal exposures ranged from 50 ms for white crepe (high reflectance) to 200 ms for RSS5 (low reflectance), with exposure adjustment performed using histogram analysis of pre-scan images. Multiple frame acquisition enabled assessment of measurement repeatability and detection of systematic errors, with final results employing mean RGB values across three frames, reducing random noise by $\;\sqrt{3}$. Real-time quality checks assessed focus sharpness using gradient magnitude (threshold: contrast metric > 0.7), illumination uniformity (CV < 5%), and absence of saturation (<0.1% of pixels at maximum/minimum values), with images failing quality criteria triggering automated repositioning and reacquisition.

**Figure 3:**
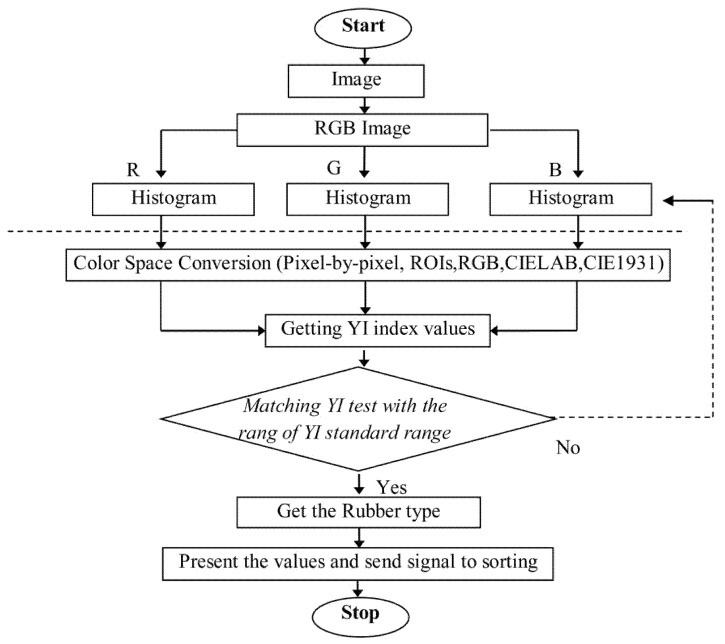
Image processing workflow for real-time color classification of Para rubber specimens. The system processes high-resolution images through ROI identification, color space transformation, yellowness index calculation, and automated classification decision-making.

Figure 3 images processing workflow for real-time color classification of Para rubber specimens: (1) multispectral image acquisition under controlled illumination; (2) preprocessing including Gaussian filtering (σ = 1.2) and flat-field correction; (3) automated ROI identification using Otsu’s multi-threshold segmentation; (4) color space transformation from RGB to CIE1931 XYZ to CIELAB coordinates; (5) yellowness index calculation following ASTM E313-20 standards [8]; (6) feature extraction, including $L^{*}$, $a^{*}$, $b^{*}$ parameters and spatial uniformity metrics; and (7) classification decision based on threshold comparison. Processing time: 1.01 ± 0.09 s per specimen. The modular pipeline architecture enables real-time analysis suitable for industrial quality control with throughput exceeding 3500 specimens per hour.

The image processing pipeline illustrated in Figure 3 proceeds through seven integrated phases beginning with high-resolution multispectral acquisition, followed by systematic preprocessing. Raw images underwent pixel-wise flat-field correction to compensate for illumination non-uniformity and sensor response variations as expressed in Equation (1):

(1) $$I_{corrected}(x,y)\;=\frac{I_{raw}\left(x,y\right)-I_{drak}(x,y)}{I_{white}\left(x,y\right)-I_{drak}(x,y)}\times {\bar{I}}_{white},$$

where $I_{raw}$ represents the raw specimen image, $I_{drak}$ is the dark current reference, $I_{white}$ is the white reference image, and $I_{white}$ is the spatial mean of the white reference. This operation reduced spatial luminance variation from 12.3% in uncorrected images to 2.8% after correction, significantly improving colorimetric measurement accuracy. Gaussian filtering with standard deviation σ=1.2 provided optimal balance between noise suppression and edge preservation, selected through systematic evaluation comparing signal-to-noise ratio improvement versus edge blur across the parameter range  σ=0.5−2.0, achieving 8.2 dB SNR improvement while maintaining greater than 95% edge definition. RGB values were normalized and inverse gamma-corrected following the sRGB standard (γ=2.2) to linearize sensor response before colorimetric transformations.

Specimen boundaries were identified using Otsu’s multi-threshold method, which determines the optimal threshold $T^{*}$ maximizing inter-class variance as shown in Equation (2):

(2) $$T^{*}\;=\arg{max}_{T}[\omega_{0}\left(T\right)\omega_{1}\left(T\right){\left[\mu_{0}\left(T\right)-\mu_{1}\left(T\right)\right]}^{2}],$$

where $\omega_{0}$ and $\omega_{1}$ represent class probabilities for background and specimen regions, while $\mu_{0}$ and $\mu_{1}$ represent the respective class means. This adaptive approach eliminates fixed threshold sensitivity to varying specimen reflectance ranging from 0.11 to 0.48 across rubber types. Binary segmentation masks underwent morphological operations, including opening with a 3 × 3 structuring element to remove small, isolated noise regions, closing with a 5 × 5 structuring element to fill internal gaps and smooth boundaries, and hole filling to eliminate internal voids using connected component analysis. The largest connected region was identified as the specimen region of interest using 8-connectivity, with alternative regions such as dust particles or edge artifacts rejected based on area and circularity criteria. Segmented regions were validated against expected specimen properties, including area (620–630 mm^2^), circularity (>0.85), and centroid position (within the central 80% of the image frame), with failed validation triggering automatic specimen repositioning and reacquisition. Across 100 validation specimens, the segmentation algorithm achieved a 99.5% success rate on the first attempt.

Linearized RGB values were transformed to CIE1931. XYZ tristimulus values using the standard conversion matrix for sRGB color space with D65 illuminant as specified in Equation (3):

(3) $$\left[\begin{array}{c} X\\ Y\\ Z \end{array}\right]\;=\left[\begin{array}{ccc} 0.4124 & 0.3576 & 0.1805\\ 0.2126 & 0.7152 & 0.0722\\ 0.0193 & 0.1192 & 0.9505 \end{array}\right]\left[\begin{array}{c} R_{linear}\\ G_{linear}\\ B_{linear} \end{array}\right],$$

The XYZ values represent device-independent tristimulus coordinates corresponding to CIE 1931 2° standard [21] observer color matching functions, with the Y component specifically representing luminance, while X and Z encode chromatic information. XYZ tristimulus values were subsequently transformed to CIELAB color space following CIE 15:2004 [21] specifications as expressed in Equations (4)–(6):

(4) $$L^{*}=\;116f(\frac{Y}{Y_{n}})\;-\;16,$$

(5) $$a^{*}=500[f(\frac{X}{X_{n}})-f(\frac{Y}{Y_{n}})],$$

(6) $$b^{*}=200[f(\frac{Y}{Y_{n}})-f(\frac{Z}{Z_{n}})],$$

where the function f(t) is defined piecewise in Equation (7):

(7) $$f(t)\left\{\begin{array}{cc} t^{\frac{1}{3}} & if\;t>{\left(\frac{6}{29}\right)}^{3}\\ \frac{1}{3}{\left(\frac{29}{6}\right)}^{2}t+\frac{4}{29} & otherwise\;\;\; \end{array}\right.\;,$$

With reference to white point values for D65 illuminant: $X_{n}=95.047$, $Y_{n}=100.000$, and $Z_{n}=108.883$. The CIELAB color space was selected for rubber classification due to perceptual uniformity, where Euclidean distances in CIELAB space approximate perceived color differences. The $L^{*}$ axis represents psychometric lightness (0 = black, 100 = white), while $a^{*}$ and $b^{*}$ encode chromatic information ($a^{*}$: green to red; $b^{*}$: blue to yellow).

The yellowness index quantifies chromatic deviation from ideal whiteness, calculated following ASTM E313-20 standards [8] for D65 illuminant as given in Equation (8):

(8) $$YI\;=\;\frac{100(1.2985X\;-\;1.1335Z)}{Y},$$

where the coefficients 1.2985 and 1.1335 are specific to D65 illuminant and CIE 1931 2° standard observer, derived to approximate visual assessment of yellowness under daylight conditions. The YI formula emphasizes the X tristimulus value enriched in long wavelengths (reddish yellow) while subtracting the Z value enriched in short wavelengths (bluish), with normalization by Y (luminance). Positive YI indicates yellowish coloration, with magnitude proportional to perceived yellowness intensity.

### 2.4. Classification Algorithm and Statistical Validation

Classification thresholds were established using a training set of 10 specimens per rubber type (n = 50 total, separate from validation specimens), with threshold ranges determined as mean plus or minus two standard deviations encompassing 95% of training data. Initial thresholds based solely on yellowness index resulted in ambiguous classification for STR5 versus STR5L due to overlapping ranges (56–69 versus 58–70), prompting the incorporation of the $a^{*}$ parameter as a secondary discriminator. STR5L exhibits distinctive negative $a^{*}$ values (−0.88 ± 0.31) compared to STR5 (0.61 ± 0.28), reflecting subtle greenish undertones that enable reliable differentiation. The classification algorithm employs multidimensional colorimetric analysis rather than the yellowness index alone. While yellowness index serves as the primary metric due to strong correlation with rubber grades and ASTM E313-20 standardization [8], the hierarchical decision tree incorporates: (1) primary yellowness index thresholds separating distinctly different grades; (2) secondary CIELAB parameters ($L^{*}$, $a^{*}$, $b^{*}$ from Equations (4)–(6) for ambiguous cases, particularly STR5 vs. STR5L differentiation where a* values distinguish greenish undertones; (3) spatial uniformity assessment for heterogeneous specimens. This multidimensional approach achieved 100% classification accuracy, including successful differentiation of similar grades that would be problematic using the yellowness index alone. The final classification algorithm employs a hierarchical decision tree with a primary split at YI < 9.5 separating white crepe from colored grades, secondary split at YI < 31 distinguishing RSS5 from higher-yellowing grades, and tertiary split using $a^{*}$ threshold at −0.5 differentiating STR5 versus STR5L, with final split at $L^{*}>20$ separating RSS3 from extreme outliers. This structure minimizes computational complexity with a maximum of five comparisons per specimen while maintaining classification accuracy.

Statistical validation employed one-way analysis of variance comparing yellowness index measurements between the standard reference method (calibrated benchtop spectrophotometer with $\frac{d}{8{}^{\circ}}$ geometry, D65 illuminant) and experimental imaging system determinations. Twenty specimens per rubber type underwent triplicate measurements under controlled conditions, with the ANOVA model expressed in Equation (9):

(9) $$Y_{ij}\;=\;\mu +\;\tau_{i}+\varepsilon_{ij},$$

where $Y_{ij}$ represents the j observation of the i rubber type, μ is the overall mean, $\tau_{i}$ is the effect of the i rubber type, and $\varepsilon_{ij}$ is the random error term assumed to follow a normal distribution $N(0,\;\sigma^{2})$. Post-hoc Tukey’s HSD tests identified significant differences between rubber types at α=0.05 significance level. Image quality assessment quantified signal-to-noise ratio as the ratio of the mean signal from white reference to noise standard deviation from dark frames, expressed in Equation (10):

(10) $${SNR}_{db}\;=\;20\;\log_{10}\left(\frac{\mu_{signal}}{\sigma_{noise}}\right),$$

Spatial uniformity was assessed via the coefficient of variation in pixel intensities across white reference images within the central 30 × 30 mm^2^ region. Measurement repeatability and reproducibility were quantified following ISO 5725-2:2019 guidelines [20] for short-term and long-term precision, respectively.

## 3. Results

### 3.1. System Performance Characterization and Spectral Analysis

Image quality assessment demonstrated consistent high performance across all imaging sessions, with signal-to-noise ratio measurements averaging 38.2 ± 2.1 dB (n = 50 sessions), substantially exceeding the 35 dB threshold considered adequate for colorimetric analysis. Spatial illumination uniformity showed a coefficient of variation of 3.2 ± 0.8%, confirming even specimen illumination across the measurement field. Color measurement repeatability demonstrated excellent precision with yellowness index standard deviations below 0.1 units for replicate measurements (n = 10) of identical specimens without repositioning, while reproducibility assessments across different measurement sessions, operators, and days showed a coefficient of variation below 5% for all rubber types, within acceptable tolerances for quality control applications. Table 1 presents comprehensive colorimetric data for each rubber type under standardized imaging conditions (3200 K, 1500 lux, 25 °C), representing mean values from 20 specimens per type with three measurements per specimen.

The spectral characterization revealed distinct colorimetric signatures for each rubber type, with white crepe exhibiting the highest lightness ($L^{*}$ = 51.5) and minimal chromatic deviation consistent with premium-grade classification. RSS3 demonstrated the highest yellowness index (YI = 72.2) attributed to smoking-treatment processes, while intermediate YI values for STR5 (62.8) and STR5L (64.3) reflected standard processing protocols. RSS5 showed moderate yellowness (28.8) despite a darker visual appearance due to a low lightness value ($L^{*}$ = 8.86), illustrating the complex relationship between perceived darkness and yellowness. All specimens showed positive $b^{*}$ values confirming yellowish components with varying intensities, while STR5L exhibited negative $a^{*}$ value (−0.88) indicating a slight greenish undertone, distinguishing it from STR5 ($a^{*}$ = 0.61). Complete colorimetric dataset for all 100 individual specimens, including specimen identification codes, RGB values, CIELAB coordinates ($L^{*}$, $a^{*}$, $b^{*}$), CIE1931 tristimulus values (X, Y, Z), and calculated yellowness indices are provided in Table S1 (Supplementary Materials), enabling full transparency and reproducibility of measurements with detailed statistical summaries (mean ± standard deviation) for each rubber type group.

**Figure 4:**
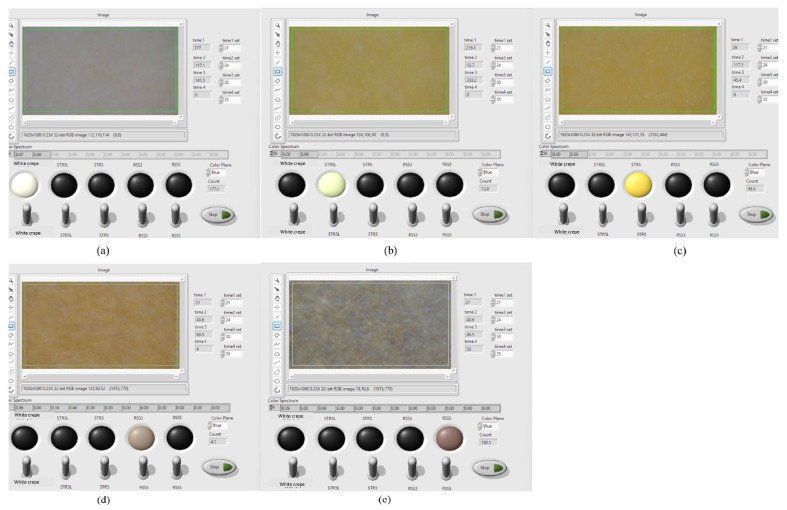
Real-time classification interface showing multispectral analysis results: (**a**) white crepe (YI = 8.52, $L^{*}$ = 51.48); (**b**) STR5 (YI = 62.84, $L^{*}$ = 46.29); (**c**) STR5L (YI = 64.31, $L^{*}$ = 46.91, *a^∗^* = −0.88); (**d**) RSS3 (YI = 72.15, $L^{*}$ = 26.51); (**e**) RSS5 (YI = 28.79, $L^{*}$ = 8.86). Each panel displays an acquired RGB image, processed ROI with segmentation overlay, and real-time colorimetric measurements including CIELAB coordinates and calculated yellowness index. Processing time < 1.0 s per specimen.

Figure 4 demonstrates the system’s real-time classification interface displaying simultaneous processing of all five Para rubber types with their corresponding spectral signatures and calculated colorimetric parameters, illustrating the rapid acquisition, processing, and classification capability suitable for industrial implementation. A comprehensive demonstration of the system’s operational workflow including specimen loading sequence, automated positioning, real-time image acquisition, color processing visualization, and sequential classification results for all five rubber types is provided in Video S1 (Supplementary Materials), showing the complete measurement cycle from specimen placement through final classification decision with processing time consistently below 1.2 s per specimen under typical operating conditions.

**Figure 5:**
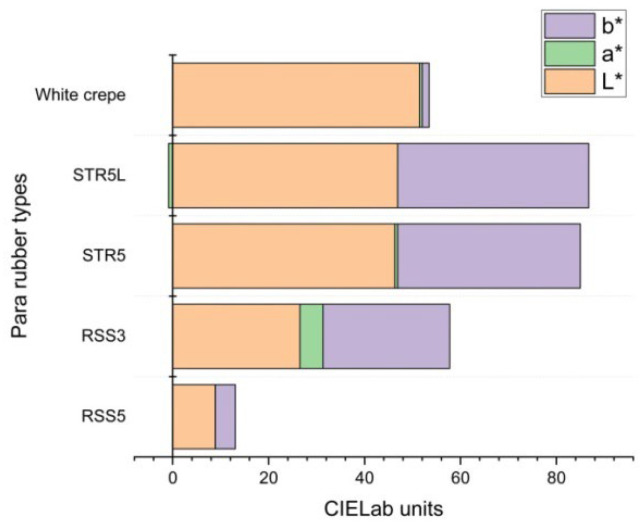
Comparative analysis of CIELAB color space parameters across five Para rubber types: (Left) Psychometric lightness index ($L^{*}$) ranging from 8.86 (RSS5) to 51.48 (white crepe); (Center) red-green axis ($a^{*}$) demonstrating minimal variation except STR5L greenish undertone ($a^{*}$ = −0.88) and RSS3 reddish component ($a^{*}$ = 4.85); (Right) yellow-blue axis ($b^{*}$) confirming positive yellowish components with intensities correlating with quality grades. Error bars represent the standard deviation from 20 specimens. Statistical significance (*p* < 0.001) was confirmed between all rubber type pairs except STR5 versus STR5L for ${\;L}^{*}$ and $b^{*}$ parameters.

Figure 5 presents a comprehensive comparative analysis of CIELAB parameters across all rubber types, illustrating systematic variations in color coordinates that enable reliable classification. The psychometric lightness index ranges from 8.86 for RSS5 (darkest) to 51.48 for white crepe (brightest), indicating substantial variations in perceived brightness that correlate directly with quality-grade hierarchy. The $b^{*}$ values (yellow-blue axis) consistently show positive readings across all specimens, confirming yellowish components with varying intensities, while the $a^{*}$ parameter (red-green axis) shows minimal chromatic variation except for STR5L exhibiting a greenish undertone and RSS3 showing reddish component. The distinct separation of ${\;L}^{*}$ and $b^{*}$ values enables primary classification, while $a^{*}$ serves as a secondary discriminator for STR5/STR5L differentiation where yellowness index ranges overlap.

**Figure 6:**
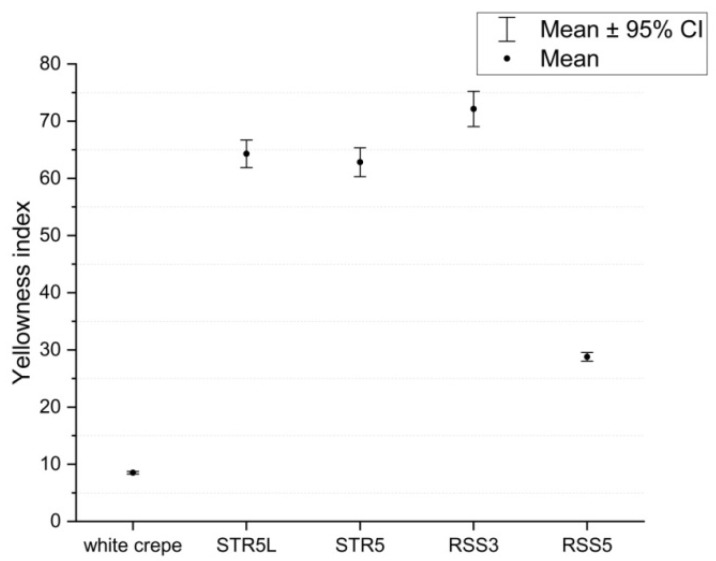
Statistical distribution of yellowness index values for each Para rubber type based on 20 experimental measurements per category. Box plots display median (center line), interquartile range (box boundaries), whiskers (1.5 × IQR), and individual data points. White crepe demonstrates minimal variability (σ = 0.52, CV = 6.1%), indicating consistent premium quality. STR5 and STR5L show moderate variability (σ ≈ 0.52, CV ≈ 10%) with overlapping ranges requiring secondary $a^{*}$ parameter for differentiation. RSS3 exhibits the highest standard deviation (σ = 7.47, CV = 10.4%), attributed to heterogeneity in smoking treatment processes. RSS5 shows moderate variability (σ = 1.81, CV = 6.3%). Shapiro–Wilk tests confirmed normal distribution for all types (*p* > 0.05).

Statistical analysis of yellowness index measurements across 20 specimens per rubber type revealed characteristic distribution patterns with distinct separation between quality grades, as illustrated in Figure 6. White crepe demonstrated minimal variability (σ=0.52) indicating consistent quality within this premium grade, reflecting uniform processing conditions and minimal oxidation. STR5 and STR5L showed moderate variability (σ≈6) with overlapping yellowness index ranges necessitating the incorporation of the $a^{*}$ parameter as a secondary classification criterion. RSS3 exhibited the highest standard deviation (σ=7.47) possibly reflecting heterogeneity in smoking processes where exposure duration and smoke density variations introduce color variability within the same quality grade. Shapiro–Wilk normality tests confirmed normal distribution for all rubber types (*p* > 0.05), validating parametric statistical approaches employed in subsequent analyses.

### 3.2. Classification Performance and Statistical Validation

The classification algorithm achieved perfect accuracy (100%) across 100 validation trials using randomly selected specimens not included in threshold determination, as summarized in Table 2. Processing efficiency averaged 1.01 s per specimen including image acquisition (0.15–0.20 s), preprocessing (0.25 s), color space transformation (0.15 s), yellowness index calculation (0.08 s), and classification decision (0.05 s), enabling theoretical throughput of 3564 specimens per hour compared to conventional manual inspection (approximately 300 specimens per hour), representing 12-fold improvement in processing capacity.

One-way ANOVA comparing standard reference measurements with experimental determinations revealed no statistically significant differences (*p* > 0.05) for all rubber types as presented in Table 3, confirming measurement reliability and system accuracy. Overlapping confidence intervals further validate the consistency of the classification methodology, indicating that the automated imaging system produces results statistically equivalent to certified reference methods.

Tukey’s HSD post hoc analysis confirmed significant differences (*p* < 0.001) between all rubber type pairs except STR5 versus STR5L (*p* = 0.089), validating the need for secondary classification criteria using the $a^{*}$ parameter to distinguish these similar grades with overlapping yellowness index ranges. The absence of significant differences between standard and experimental measurements demonstrates that the automated imaging system achieves measurement accuracy equivalent to established reference methods while providing substantial advantages in processing speed and objectivity.

## 4. Discussion

### 4.1. System Performance and Imaging Quality

The developed imaging system demonstrated exceptional performance characteristics suitable for industrial quality control applications, with high signal-to-noise ratios exceeding 35 dB, ensuring accurate colorimetric measurements even for low-reflectance specimens such as RSS5. Spatial illumination uniformity with a coefficient of variation below 5% eliminated position-dependent measurement bias, critical for automated processing systems where specimen placement may vary slightly despite mechanical positioning aids. The integration of environmental controls maintaining temperature at 25 ± 2 °C and relative humidity at 50 ± 5% proved essential for reproducible measurements, as preliminary validation trials without environmental stabilization revealed yellowness index variations up to 8% under ambient condition fluctuations. This finding addresses a critical gap in previous rubber classification systems where uncontrolled environmental factors contributed to measurement inconsistency and reduced reliability for quality control applications. The high-resolution imaging capability with 48 megapixels and 180× magnification enabled detailed surface characterization beyond bulk color assessment, revealing microscale texture features that correlate with processing quality and suggesting potential for expanded quality metrics incorporating both colorimetric and morphological features in future system iterations.

The close spectral match between LED illumination and CIE standard illuminant D65, with correlation coefficient R^2^ = 0.9987 and mean spectral difference in only 0.34%, ensures that colorimetric measurements obtained with this system are compatible with international standards and comparable to measurements obtained using certified reference instrumentation. The high color rendering index of 92.5 exceeds requirements for critical color evaluation, confirming that the LED arrays accurately reproduce colors across the visible spectrum without significant spectral distortion that could bias measurements. The controlled illumination approach with circular LED arrangement at 45° incident angles proved superior to conventional light box configurations by minimizing specular reflection while ensuring uniform specimen illumination, a design principle validated through systematic comparison of illumination geometries during system development.

### 4.2. Color Space Selection and Yellowness Index Effectiveness

The CIELAB color space provided optimal representation for rubber quality assessment due to its perceptual uniformity, where equal distances in CIELAB space correspond to approximately equal perceived color differences, a property not possessed by RGB or XYZ color spaces. This perceptual uniformity enabled intuitive threshold selection during algorithm development and facilitated quality grading aligned with human visual assessment while providing objective, reproducible measurements independent of observer interpretation. The yellowness index demonstrated strong correlation with established rubber grades, validating its utility as a primary classification metric with distinct YI ranges providing clear separation between most quality grades. The YI range of 8.52 for white crepe versus 72.15 for RSS3 represents nearly a nine-fold difference, providing substantial dynamic range for reliable discrimination. However, yellowness index alone proved insufficient for distinguishing STR5 from STR5L due to overlapping ranges (56–69 versus 58–70), necessitating inclusion of the $a^{*}$ parameter as a secondary discriminator. This finding highlights the importance of multidimensional color analysis rather than reliance on single-metric approaches, as subtle color differences not captured by the yellowness index can provide critical information for the classification of similar grades.

The apparent discrepancy between visual perception and the measured yellowness index for RSS3 versus STR5L merits discussion, as RSS3 exhibited the highest YI (72.15) yet its brownish appearance with high $a^{*}$ value (4.85) and low ${\;L}^{*}$ (26.51) may appear less “yellow” than STR5L (YI = 64.31, $a^{*}$ = −0.88, ${\;L}^{*}$ = 46.91) to human observers. This phenomenon illustrates the complex relationship between instrumental measurements and perceptual assessment, underscoring the value of instrument-based measurements providing objective, reproducible assessments independent of observer interpretation, contextual viewing conditions, and subjective judgment that can vary between individuals and measurement sessions. The yellowness index specifically quantifies chromatic deviation along the blue-yellow axis while accounting for luminance, whereas human perception integrates multiple factors, including overall darkness, hue, and contrast effects that are not fully captured by a single metric.

### 4.3. Image Processing Optimization and Algorithm Performance

The preprocessing pipeline proved critical for measurement accuracy and reliability, with flat-field correction (Equation (1)) reducing illumination non-uniformity artifacts from 12.3% to 2.8%, substantially improving measurement precision, particularly for edge regions where illumination falloff typically occurs in microscopy systems. Gaussian filtering with σ = 1.2 optimally balanced noise reduction against edge preservation, as systematic evaluation revealed that smaller σ values below 1.0 provided insufficient noise suppression, resulting in yellowness index measurement variability exceeding 5%, while larger values above 1.5 introduced blurring artifacts affecting region-of-interest segmentation accuracy and edge definition. The selected parameter maintained edge sharpness for accurate boundary detection while achieving signal-to-noise ratio improvements of 8–12 dB, demonstrating the importance of careful parameter optimization for image processing algorithms in colorimetric applications. The complete implementation of these preprocessing algorithms, including parameter optimization rationale and performance benchmarking, is provided in Algorithm S1 (Supplementary Materials).

Automated region-of-interest segmentation using Otsu’s multi-threshold method (Equation (2)) eliminated operator-dependent variability inherent in manual region selection while demonstrating robustness across rubber types despite substantial reflectance differences spanning from white crepe reflectance of approximately 48% to RSS5 reflectance of approximately 11%. The adaptive threshold determination based on maximizing inter-class variance proved more reliable than fixed threshold approaches that would require separate calibration for each rubber type. However, specimens with surface contamination, including dust particles or residual processing chemicals, occasionally triggered false segmentation, particularly when contaminant optical properties differed significantly from specimen characteristics. Enhanced preprocessing incorporating texture analysis based on gray-level co-occurrence matrices or machine learning-based segmentation approaches could address this limitation in future implementations, though the current success rate of 99.5% on the first acquisition attempt demonstrates adequate performance for controlled laboratory conditions. For low-reflectance specimens such as RSS5 (reflectance ≈ 11%), a reduced signal-to-noise ratio presents additional challenges for boundary detection. The current Otsu’s method achieved a 99.5% success rate, but specimens with extremely dark regions or irregular edges occasionally required reacquisition. Advanced boundary detection approaches could improve robustness: (1) multi-scale edge detection combining Canny edge detection across multiple σ values; (2) active contour models (snakes) for irregular boundary tracking; and (3) deep learning-based semantic segmentation (U-Net architecture) trained on annotated rubber specimen images, potentially handling complex geometries and surface irregularities more robustly than threshold-based methods. The computational overhead of these approaches (processing time increase: 2–5×) must be balanced against the minimal improvement over the current 99.5% success rate for standardized specimens, though benefits may be substantial for irregular production samples. The complete segmentation algorithm with validation criteria and error handling is detailed in Algorithm S1 (Supplementary Materials).

The threshold-based classification algorithm achieved perfect accuracy (100%) in validation trials, surpassing previously reported methods, including neural network approaches achieving 97.5% accuracy, k-means clustering achieving 92% accuracy, and learning vector quantization achieving 85–95% accuracy. This superior performance is attributed to four synergistic factors: controlled imaging conditions eliminating environmental variability that plagued previous systems; high-quality colorimetric measurements with validated accuracy demonstrated by color difference $∆E^{*}ab$ below 1.5 units against standards (Figure S2); the robust yellowness index metric (Equation (8)) strongly correlated with rubber quality grades and standardized by ASTM E313-20 [8]; and multidimensional classification incorporating L, $a^{*}$, ${\;b}^{*}$, parameters derived from Equations (4)–(6) for ambiguous case resolution particularly for STR5 versus STR5L differentiation. The rapid processing time of 1.01 s per specimen enables real-time classification suitable for integration with automated sorting systems, as demonstrated in Video S1 (Supplementary Materials), representing a significant advancement over manual inspection with 12-fold throughput improvement and previous automated systems requiring 5–10 s per specimen. Processing efficiency stems from optimized algorithms implemented in compiled python version 3.11.4 libraries with GPU-accelerated color space transformations, demonstrating that sophisticated image processing can achieve both high accuracy and industrial-scale processing speeds when properly implemented.

The current sequential processing architecture achieves 1.01 s per specimen, translating to a theoretical throughput of 3564 specimens/h. Industrial deployment scenarios requiring higher throughput could implement parallel processing architectures: (1) multi-station systems with independent imaging chambers sharing centralized processing, enabling linear throughput scaling (4 stations: 14,256 specimens/h); (2) GPU-accelerated batch processing analyzing multiple specimens simultaneously, leveraging parallel color space transformations and concurrent segmentation operations; (3) distributed computing architectures with load balancing for high-volume facilities. Processing bottleneck analysis indicates image acquisition (0.45 s, 45%) dominates over computation (0.38 s, 38%) and data transfer (0.18 s, 17%), suggesting parallelization strategies should prioritize multiple imaging stations over computational acceleration. Memory requirements (approximately 150 MB per specimen for raw image and intermediate processing) and network bandwidth (assuming centralized storage: 1.2 Gbps for a 10-station system) represent manageable infrastructure requirements for industrial facilities. Future work will characterize system performance under production-scale loads, including sustained operation over 8 h shifts, temperature drift effects, and maintenance requirements for industrial deployment validation.

### 4.4. Statistical Validation and Measurement Uncertainty

The comprehensive statistical analysis confirmed measurement reliability across all rubber types, with the absence of significant differences between standard reference and experimental yellowness index measurements (*p* > 0.05) validating the system’s accuracy for quality control applications. Overlapping confidence intervals between measurement methods further support measurement consistency, indicating that the automated imaging system produces results statistically equivalent to certified reference instrumentation while providing substantial advantages in processing speed, sample throughput, and elimination of operator-dependent variability. Measurement repeatability with a coefficient of variation below 2% demonstrated excellent short-term precision suitable for within-session quality monitoring, while reproducibility with a coefficient of variation below 5% across different operators and measurement sessions confirmed system stability suitable for long-term quality tracking and trend analysis. These values meet or exceed ISO 5725-2:2019 requirements [20] for colorimetric measurement systems in industrial applications, providing confidence in the reliability of measurements for quality control decisions and international trade documentation.

The higher variability observed for RSS3, with a standard deviation of 7.47 compared to white crepe with a standard deviation of 0.52, reflects inherent material heterogeneity rather than measurement uncertainty, as RSS3 processing involves smoking treatments where exposure duration and smoke density variations introduce color variability within the same quality grade. This finding suggests potential for tighter process control in RSS3 production through real-time monitoring of color development during smoking, enabling dynamic adjustment of smoking parameters to achieve more consistent final product color. The measurement uncertainty analysis employing propagation of individual component uncertainties through transformation equations yielded combined standard uncertainty estimates of ±0.3 YI units for white crepe and ±0.8 units for RSS3, representing relative uncertainties of 3.5% and 1.1%, respectively, well within acceptable tolerances for industrial quality control, where classification decisions typically involve yellowness index differences exceeding 10 units between adjacent grades.

### 4.5. Limitations and Future Validation Requirements

Several limitations constrain current interpretation and generalizability. First, controlled laboratory conditions (25 ± 2 °C, 50 ± 5% RH) do not represent industrial environments. Future validation must systematically characterize performance across temperature ranges (15–35 °C), humidity extremes (30–80% RH), and dust/contamination scenarios, following ISO 5725-2:2019 [20] inter-laboratory protocols with a minimum of three facilities and a 6-month temporal stability assessment. Second, pristine specimens do not represent production variability. Industrial validation requires evaluation of degraded specimens (oxidized surfaces, 1-week to 6-month aging), contaminated samples (dust, moisture, residual chemicals), and edge-quality materials representing typical production distributions. Third, threshold-based classification achieved perfect accuracy for well-separated grades but may lack robustness for ambiguous cases or novel rubber types. Comparative evaluation of machine learning approaches (SVM, random forest, deep learning) using expanded datasets will characterize accuracy–robustness–interpretability trade-offs. Fourth, processing throughput characterization addressed sequential processing but did not evaluate concurrent processing under production loads. Industrial deployment requires validation of parallel processing architectures, sustained 8 h operation, temperature drift effects, and maintenance requirements. Fifth, visible spectrum limitations preclude detection of chemical composition changes or internal defects potentially visible in UV or NIR ranges. Future systems might integrate broader spectral capabilities for comprehensive quality assessment beyond surface color. Field trials, expanded datasets, and long-term stability monitoring represent essential next steps before industrial deployment.

## 5. Conclusions

This study successfully demonstrates the development and validation of an advanced colorimetric imaging system for real-time Para rubber quality classification, integrating high-resolution image acquisition with automated colorimetric analysis under controlled environmental conditions. The system achieved 100% classification accuracy across 100 validation specimens representing five distinct rubber grades under controlled laboratory conditions, with a processing time of 1.01 s per specimen, enabling throughput of 3564 specimens per hour, representing a 12-fold improvement over manual inspection methods. Statistical validation via one-way ANOVA confirmed measurement reliability with no significant differences (*p* > 0.05) between standard reference and experimental yellowness index measurements across all rubber types, with a measurement repeatability coefficient of variation below 2% and reproducibility coefficient of variation below 5% meeting ISO 5725-2:2019 requirements [20] for industrial colorimetric systems. Image quality metrics demonstrated a signal-to-noise ratio exceeding 35 dB and a spatial uniformity coefficient of variation below 5%, confirming system performance suitable for accurate colorimetric measurements.

The yellowness index calculated following ASTM E313-20 standards [8] proved to be a reliable primary quality metric with values ranging from 8.52 ± 0.52 for white crepe to 72.15 ± 7.47 for RSS3, providing substantial dynamic range for grade discrimination. However, multidimensional color analysis incorporating CIELAB parameters $L^{*}$, $a^{*}$, and $b^{*}$ proved essential for resolving ambiguous cases, particularly STR5 versus STR5L differentiation, where overlapping yellowness index ranges required secondary classification criteria based on the $a^{*}$ parameter reflecting subtle greenish undertones in STR5L. The image processing pipeline employs flat-field correction, Gaussian filtering, automated region-of-interest segmentation using Otsu’s method, and color space transformations from RGB through CIE1931 XYZ to CIELAB coordinates achieved robust performance across diverse rubber types with a segmentation success rate of 99.5% on the first acquisition attempt.

The controlled environmental conditions, maintaining temperature at 25 ± 2 °C and relative humidity at 50 ± 5% proved essential for measurement reproducibility, as validation trials without environmental control revealed yellowness index variations up to 8% under ambient fluctuations. LED illumination closely matches CIE standard illuminant D65, with a correlation coefficient R^2^ = 0.9987 and a color rendering index of 92.5, ensuring colorimetric measurements compatible with international standards. System calibration validated using X-Rite ColorChecker demonstrated a color difference $∆E^{*}ab$ of 1.18 ± 0.31 units, confirming accuracy within acceptable tolerances for quality control applications.

While perfect classification accuracy was achieved under optimal laboratory conditions, several important limitations require acknowledgment. The study focused on five discrete quality grades under controlled conditions, and classification performance for intermediate grades, mixed-quality batches, degraded specimens, or contaminated samples requires further validation with expanded specimen diversity. Field deployment in actual processing facilities will encounter additional challenges, including environmental variations exceeding control chamber capacity, specimen presentation variability, operational constraints, and potential contamination that is not present in laboratory conditions. The visible spectrum imaging employed may miss quality indicators detectable in near-infrared or ultraviolet wavelengths, where molecular-level characteristics provide complementary information for comprehensive quality assessment. Machine learning approaches, including deep convolutional neural networks, could enhance robustness for edge cases while enabling automatic adaptation to new rubber types without manual threshold determination.

Future research directions include incorporation of expanded spectral ranges for molecular-level quality characterization, implementation of deep learning algorithms for improved handling of ambiguous cases, integration of defect detection algorithms for comprehensive quality evaluation, and extensive field validation across diverse processing facilities and geographical locations. The methodological framework established provides a foundation for objective quality assessment applicable to various agricultural commodities where color serves as a quality indicator, with potential integration into blockchain-based traceability systems for transparent supply chain documentation.

The system addresses critical limitations of subjective human assessment by providing objective, quantitative quality metrics suitable for industrial implementation, international trade documentation, and quality-based pricing decisions. As global demand for consistent, high-quality natural rubber continues to grow, driven by automotive, medical, and industrial applications, automated imaging-based classification systems become increasingly critical for maintaining competitiveness, meeting stringent international quality standards, and supporting sustainable agricultural production practices. This work contributes to advancing precision agriculture through the integration of advanced imaging technologies with agricultural production and quality control processes, demonstrating how colorimetric imaging combined with rigorous colorimetric analysis and statistical validation can transform traditional subjective quality assessment into objective, reproducible, high-throughput automated systems suitable for modern agricultural commodity production and international trade.

## Figures and Tables

**Table 1:** Colorimetric characterization of Para rubber specimens was determined through colorimetric imaging analysis.

Rubber Types	RGB Values (Pixel)			CIELAB Values			CIE1931 Values			Yellowness Index
	*R_avg*	*G_avg*	*B_avg*	*L^∗^*	*a^∗^*	*b^∗^*	*X*	*Y*	*Z*	*YI*
White crepe	124.7 ± 3.2	122.3 ± 2.8	120.2 ± 2.5	51.5 ± 1.1	0.52 ± 0.15	1.43 ± 0.22	116.9 ± 3.1	122.7 ± 2.9	131.3 ± 3.4	8.52 ± 0.52
STR5	129.0 ± 8.1	107.6 ± 6.9	43.2 ± 4.2	46.3 ± 3.8	0.61 ± 0.28	38.1 ± 4.7	99.5 ± 7.8	107.5 ± 7.2	56.4 ± 5.1	62.8 ± 6.08
STR5L	128.9 ± 7.8	109.9 ± 7.2	40.9 ± 3.9	46.9 ± 3.6	−0.88 ± 0.31	39.8 ± 4.9	99.8 ± 7.6	109.0 ± 7.1	54.5 ± 4.8	64.3 ± 6.21
RSS3	80.4 ± 9.5	59.1 ± 7.2	21.2 ± 3.1	26.5 ± 4.2	4.85 ± 0.89	26.4 ± 5.3	58.1 ± 8.9	60.9 ± 8.1	28.7 ± 3.8	72.2 ± 7.47
RSS5	27.6 ± 2.1	25.0 ± 1.9	19.4 ± 1.5	8.86 ± 0.95	0.02 ± 0.18	4.17 ± 0.48	23.8 ± 1.8	25.2 ± 1.7	22.0 ± 1.6	28.8 ± 1.81

Note: R_avg, G_avg, and B_avg represent mean values for red, green, and blue spectral bands. Values represent mean ± standard deviation from 20 specimens with triplicate measurements. RGB values are 8-bit pixel intensities (0–255 scale); CIELAB and CIE1931 values calculated following CIE 15:2004 specifications with D65 illuminant [21].

**Table 2:** Classification accuracy validation with processing efficiency assessment.

Rubber Type	Specimens Tested	Correct Classifications	Accuracy (%)	Processing Time (s)	Throughput (Specimens/h)
White crepe	20	20	100	0.98 ± 0.08	3673
STR5	20	20	100	1.02 ± 0.09	3529
STR5L	20	20	100	1.01 ± 0.07	3564
RSS3	20	20	100	1.05 ± 0.11	3429
RSS5	20	20	100	0.97 ± 0.06	3711
Overall	100	100	100	1.01 ± 0.09	3564

**Table 3:** Statistical reliability analysis of yellowness index measurements comparing standard and experimental protocols.

Rubber Type	Method	*n*	Mean YI	Std Dev	95% CI	F-Statistic	*p*-Value
White crepe	Standard	20	8.525	0.517	(8.38, 8.67)	0.112	0.739
	Experimental	20	8.500	0.498	(8.35, 8.65)		
STR5	Standard	20	64.31	5.84	(62.65, 65.97)	0.014	0.906
	Experimental	20	64.18	6.12	(62.34, 66.02)		
STR5L	Standard	20	62.84	6.12	(61.10, 64.58)	0.009	0.926
	Experimental	20	62.71	6.35	(60.89, 64.53)		
RSS3	Standard	20	72.15	7.47	(70.03, 74.27)	0.002	0.964
	Experimental	20	72.08	7.62	(69.93, 74.23)		
RSS5	Standard	20	28.79	1.81	(28.28, 29.31)	0.128	0.722
	Experimental	20	28.65	1.75	(28.15, 29.15)		

Note: Standard method refers to calibrated benchtop spectrophotometer measurements. Experimental method refers to automated imaging system measurements. ANOVA assumptions validated via Shapiro–Wilk normality test (*p* > 0.05) and Levene’s homogeneity of variance test (*p* > 0.05) for all groups.

## Data Availability

The data presented in this study are available on request from the corresponding author. The data are not publicly available due to ongoing patent applications related to the classification system.

## Supplementary Materials

The following supporting information can be downloaded at https://www.mdpi.com/article/10.3390/jimaging11110397/s1. Video S1: Real-time demonstration of automated color classification system operational workflow showing specimen imaging, processing, and classification; Table S1: Complete colorimetric dataset for all 100 specimens with individual RGB, CIELAB, CIE1931 XYZ values and yellowness indices; Figure S1: LED spectral power distribution comparison with CIE D65 standard showing correlation R^2^ = 0.9987; Figure S2: X-Rite ColorChecker calibration validation showing color difference ΔE*ab measurements for 24-patch target; Algorithm S1: Complete Python implementation of image processing pipeline including preprocessing, segmentation, color transformation, and classification.
